# *Streptomyces* Strains Promote Plant Growth and Induce Resistance Against *Fusarium verticillioides* via Transient Regulation of Auxin Signaling and Archetypal Defense Pathways in Maize Plants

**DOI:** 10.3389/fpls.2021.755733

**Published:** 2021-11-25

**Authors:** Trang Minh Tran, Maarten Ameye, Frank Devlieghere, Sarah De Saeger, Mia Eeckhout, Kris Audenaert

**Affiliations:** ^1^Laboratory of Applied Mycology and Phenomics, Department of Plants and Crops, Faculty Bioscience Engineering, Ghent University, Ghent, Belgium; ^2^Laboratory of Applied Mycology, Department of Food Technology, Safety and Health, Faculty of Bioscience Engineering, Ghent University, Ghent, Belgium; ^3^Research Unit Food Microbiology and Food Preservation, Department of Food Technology, Safety and Health, Faculty of Bioscience Engineering, Ghent University, Ghent, Belgium; ^4^Center of Excellence in Mycotoxicology and Public Health, Department of Bioanalysis, Faculty of Pharmaceutical Sciences, Ghent University, Ghent, Belgium; ^5^Research Unit of Cereal and Feed Technology, Department of Food Technology, Safety and Health, Faculty of Bioscience Engineering, Ghent University, Ghent, Belgium

**Keywords:** auxin, fumonisins, *Fusarium* ear rot (FER), maize, mycotoxins, *Streptomyces*, Vietnam

## Abstract

Driven by climate change, *Fusarium* ear rot (FER) caused by *Fusarium verticillioides* occurs frequently in maize worldwide. In parallel, legislative regulations and increasing environmental awareness have spurred research on alternative FER biocontrol strategies. A promising group of bacterial control agents is *Streptomyces* species due to their metabolic versatility. However, insights into the molecular modes of action of these biocontrol agents are often lacking. This study aims at unraveling the biocontrol efficacy of *Streptomyces* rhizobacterial strains against *F. verticillioides*. We first assessed the direct antagonism of four *Streptomyces* strains ST02, ST03, ST07, and ST08. Then, a profile of 16 genes associated with intrinsic plant defense signaling was assessed in maize plants. Both *in vitro* and *in vivo* data showed that the biocontrol strain ST03 perfectly suppressed the growth of *F. verticillioides*. High inhibition efficacy was also observed for extracellular compounds in the supernatant secreted by this strain. Especially, for maize cobs, the biocontrol strain ST03 not only inhibited the proliferation of *F. verticillioides* but also significantly repressed fungal fumonisin production 7 days after inoculation. On maize plants, the direct antagonism was confirmed by a significant reduction of the fungal DNA level in soils when co-applied with *F. verticillioides* and strain ST03. In terms of its action on plants, strain ST03 induced downregulation of auxin responsive genes (*AUX1*, *ARF1*, and *ARF2*) and gibberellic acid (GA)-related gene *AN1* even in the absence of *F. verticillioides* at early time points. In leaves, the biocontrol strain induced the expression of genes related to salicylic acid (SA), and 2,4-dihydroxy-7-methoxy-1,4-benzoxazin-3-one (DIMBOA)-mediated pathways, and pathogenesis-related proteins in the presence or absence of the pathogen. Interestingly, the biocontrol strain significantly promoted plant growth even in the presence of *F. verticillioides*. All of which demonstrated that the *Streptomyces* strain ST03 is a promising FER biocontrol and a growth-promoting candidate.

## Graphical Abstract

*Streptomyces* strains promote plant growth and induce resistance against *Fusarium verticillioides* via transient regulation of auxin signaling and archetypal defense pathways in plants.

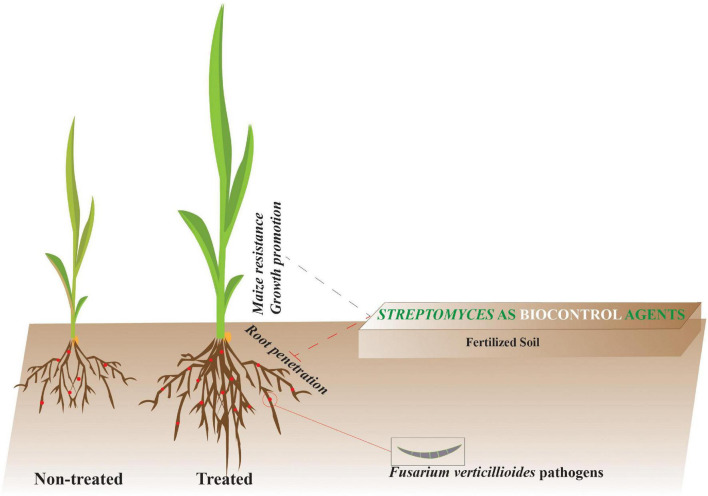

## Introduction

*Fusarium verticillioides* is a hemibiotrophic pathogen and the primary causal agent of the disease *Fusarium* ear rot (FER) in maize worldwide (Bacon et al., 2008). The disease not only reduces the productivity of maize and the nutritive value of grains, but also exerts a severe impact on human and animal health since grains infected with *F. verticillioides* can be contaminated with its toxic secondary metabolites, particularly fumonisin B_1_ (FB_1_), FB_2_, and FB_3_ (Pitt and Miller, 2016). Vulnerable parts of the maize cob such as silks (Reid et al., 1999) and insect feeding sites are the main points of entry for this pathogen (Parsons and Munkvold, 2010; Blandino et al., 2015). In some cases, the presence of *F. verticillioides* can be traced back to the seedling stage during which the fungus infects and resides asymptomatically followed by moving upwards toward the cob during anthesis and ultimately leading to FER (Oren et al., 2003; Suárez-Moreno et al., 2019). To date, no registered FER-resistant maize inbred lines are available on the market.

In the context of increasing plant disease pressure, plant pathologists face a challenging decision between the continuity of using fungicides and negative side-effects of the use of agrochemicals in crop protection (Lamichhane et al., 2016). Overreliance on fungicides is posing a threat to the environment, soil ecosystems, and human health. Therefore, research has turned to sustainable and eco-friendly alternative control measures. Rhizobacteria *Streptomyces* spp. Are well-known as not only plant growth-promoting rhizobacteria (PGPR) but also promising biocontrol agents, resulting in increased yields of agricultural crops (Dimkpa et al., 2009). From a crop protection point of view, *Streptomyces* spp. have been demonstrated to be effective against a broad spectrum of fungal phytopathogens, e.g., *Fusarium* tomato wilt (Abbasi et al., 2019), *Fusarium* head blight (FHB) (Tan et al., 2020), *Fusarium* banana wilt (Zhu et al., 2020), *Ralstonia* tomato wilt (Shen et al., 2021), and cucumber *Phytophthora* damping-off (Sadeghi et al., 2017). The effectiveness of the biocontrol can be attributed to the metabolic diversity of anti-fungal compounds and antibiotics, and their bioactive and adaptable capability in the agro-ecological relevant niche of the soil (Tan et al., 2020). Together with direct antagonism against the pathogens, several Rhizobacteria can interact with the plant defense system. They have been shown to trigger the salicylic acid (SA)- and/or jasmonic acid (JA)/ethylene (ET) mediated defense pathways in plants (Conn et al., 2008; Kurth et al., 2014; Tan et al., 2020; Vergnes et al., 2020).

Developing a biocontrol agent (BCA) for FER management requires an extensive understanding of the molecular mechanisms involved in the tripartite interaction between plant, pathogen, and BCA. A thorough understanding of phytohormone-mediated plant defense response is needed. The key phytohormones involved in the plant defense response are salicylic acid (SA), jasmonic acid (JA), ethylene (ET), abscisic acid (ABA), and auxin (Aux) (Tzin et al., 2017). Pathogenesis-related proteins (PRs) [e.g., *PR1*, and *PR10*, 1,3-β-glucanase (*PR2*), chitinase (*PR3*)] (Nasser et al., 1990), and benzoxazinoids (BXs) e.g., 2-4-dihydroxy-7-methoxy-1,4-benzoxazin-3-one (DIMBOA) (Ding et al., 2015) are responsible for plant resistance against pathogens.

To the best of our knowledge, there is little information available on the biological control of FER in maize by *Streptomyces.* Therefore, this current study aims to (i) unravel the antagonistic effect of *Streptomyces* strains to *F. verticillioides* and (ii) further insight into the molecular modes of action of the biocontrol strain in the induction of plant resistance against this fungal phytopathogen.

## Materials and Methods

### Preparation of *Fusarium* and *Streptomyces* Strains

In this study, an *F. verticillioides* strain F01.12 (accession number MZ559332) isolated by Tran et al. (2021a) was used. In addition, five other *Fusarium* strains comprising *F. proliferatum* and *F. mangiferae* were isolated from maize grains in the central highlands of Vietnam (Tran et al., 2021b), *F. oxysporum*, and *F. solani* isolated from onion in Dalat, Vietnam (Le et al., 2020) and *F. graminearum* PH1 isolated from wheat (Gale et al., 2005) were also used. Each fungus was cultured on a PDA plate (Potato Dextrose Agar, 40 g L^–1^) (Sigma Aldrich, Overijse, Belgium) for 7 days at 25°C before performing assays. To prepare a spore suspension of *F. verticillioides*, we placed a 7-day-old PDA plate of this fungus into a cabinet equipped with near-UV lights (12 h light/12 h darkness) for 7 days. The spore suspension was then collected and diluted with sterile water until a final concentration of 10^7^ conidia mL^–1^.

We used four rhizobacterial strains ST02, ST03, ST07, and ST08 isolated from soil in Ben Tre, Vietnam. For the identification of these strains, we amplified the recombinase A (recA) gene by using a primer pair of *recAF* and *recAR* as described by Guo et al., 2008 (Supplementary Table 1). The sequences were deposited to the NCBI gene bank, representing MZ614615 for strain ST02, MZ614616 for strain ST03, MZ614619 for strain ST07, and MZ614615 for strain ST08. For inoculation, each strain was grown on a TSA plate (Tryptic Soy Agar, 30 g L^–1^) (Sigma Aldrich, Overijse, Belgium) for 5 days. Three rhizobacterial colonies were then transferred into a 50-mL Falcon tube containing 20 mL TSB (tryptic soy broth, 30 g L^–1^) (Sigma Aldrich, Overijse, Belgium) and incubated for 7 days at 200 g, and 28°C. The Falcon tubes were then centrifuged at 10000 *g* for 10 min and the cell suspension was obtained by discarding the supernatant through a four-fold Mira cloth followed by re-suspending the cell pellets in 5 mL sterile water. A ten-fold serial dilution was made to determine the colony-forming unit (CFU) of each cell suspension (5 × 10^6^ CFU mL^–1^). The cell-free supernatant was concentrated 10 times using a nitrogen flow at 1 atm in a water bath at 40°C for 2 h.

### *In vitro* Antagonistic Assay on Agar Medium

In agar plates, we used two different models: (1) a diffusion assay and (2) a volatile assay. For the diffusion assay, we used a four-hole TSA plate (Φ 90 mm) (Supplementary Figure 1A). In each plate, a 7-day-old agar plug of each pathogen (Φ 5 mm) was placed in the center, and 50 μL of each rhizobacterial cell suspension or supernatant was filled in each hole. The plate was incubated at 28°C and the radius of the fungal colony was measured. In addition, to evaluate the effects of the volatile compounds produced by rhizobacterial strains we performed the volatile assay in which a TSA plate was divided into two parts by making an agar-free channel in the middle (Φ 5 mm) (Supplementary Figure 1B). Water and blank TSB were used as control.

### *In vitro* Antagonistic Assay on Liquid Medium

In this assay, we used a six-well plate containing 4.5 mL TSB per well (Supplementary Figure 1C). A ST03 cell suspension (5 × 10^7^ CFU mL^–1^) or a 10-time-concentrated supernatant was injected to each well at different volumes (μL) of 0 (0%), 31.25 (0.6%), 62.5 (1.3%), 125 (2.5%), 250 (5%), and 500 μL (10%). TSB was subsequently added to each well till 5 mL. A 7-day-old agar plug of *Fusarium verticillioides* (Φ 5 mm) was then transferred to each well. The plate was inoculated at 28°C for 7 days. To analyze the fungal growth on the surface, an image was taken using a custom-build multispectral phenotyping platform as described by Tan et al. (2020). This platform is equipped with a 6 Mp-16 bit camera mounted on a Cartesian coordinate robot and allows 6-μm-high-resolution multispectral imaging. The multispectral camera can visualize the surface growth of the fungus. Finally, biomass from each well was collected followed by freeze-dried and weighted.

### *In vivo* Antagonistic Assay on Maize Cobs

This bioassay was employed using baby maize cobs (70 mm) (Baby corn, Excel Fruits, Thailand). In each treatment, five maize cobs were disinfected with 1% NaOCl solution, washed twice with sterile water, and then placed in a one-well plate equipped with a moist Whatman paper (Supplementary Figure 1D). Subsequently, we made a hole in the middle of each cob (depth ∼ 10 mm) using a sterile 10 μL pipette tip. A mixture consisting of 5 μL spore suspension of *Fusarium verticillioides* (10^7^ conidia mL^–1^) and 5 μL cell suspension (5 × 10^6^ CFU mL^–1^) [FV + ST03 treatment)/or cell-free supernatant of rhizobacterial strain ST03 (FV + Sup) treatment] was loaded to each hole. Each maize ear was inoculated at 28°C for 7 days. Five infected maize cobs were treated with sterile water as a positive control (FV + W) and five *F. verticillioides* non-infected cobs were treated with sterile water as a negative control (W + W). The cobs were then divided into two equal parts using a scalpel. To determine the damage level caused by the pathogen, we analyzed images taken by the multispectral camera using the RGB module. Finally, each maize cob was thoroughly ground using liquid nitrogen for quantification of FB_1_, FB_2_, and FB_3_ by an LC-MS/MS method (De Boevre et al., 2012).

### *In vivo* Antagonistic Assay on Maize Seedlings

In this assay, we used two hybrid maize lines *Bt/GT* NK7328 (supplied by Syngenta company, Vietnam) and CP888 (supplied by C. P. company, Vietnam) as they are predominantly planted in the central highlands of Vietnam (Tran et al., 2021c). Maize seeds were disinfected with 1% NaOCl solution and germinated in a rectangular plastic pot (20 × 16 × 6 cm) with vermiculite substrate (Vermex, Soprema, Belgium) for 5 days. For the biocontrol treatment (FV + ST03 treatment), five germinated seeds were infected with *F. verticillioides* by soaking in a fungal suspension (10^7^ conidia mL^–1^) for 1 h, and then sowed in five glass tubes (a seed per a tube) containing soil inoculated with the ST03 cell suspension. To inoculate the soil with *Streptomyces*, 3 g polymer gel (DCM Aquaperla, Grobbendonk, Belgium) absorbed in 300 mL tap water was mixed with 2 kg non-sterile fine sand (Voss chemie-Benelux, Lier, Belgium) (De Zutter et al., 2021). This mixture was then inoculated with the ST03 cell suspension in a concentration of 10^6^ CFU g^–1^ soil 3 days before planting. The positive control comprised five *F. verticillioides* infected seeds in soil non-inoculated with the ST03 cell suspension (FV + W treatment). For the negative control, five mock seeds treated with sterile water were planted in non-inoculated soil with the strain ST03 (W + W treatment), and five mock seeds were planted in soil pre-inoculated with ST03 (W + ST03 treatment). Every 2 days plants were watered with 10 mL tap water. To assess the direct antagonism of strain ST03, the soil was collected after 2 weeks of planting and the DNA levels of *F. verticillioides* were qualified as described below.

To measure the impact of ST03 on the plant growth when co-applied with *F. verticillioides* in maize seedlings, the length and fresh biomass of leaves and roots were measured at 16 days after inoculation (dai). Chlorophyll Index (ChlIdx) was also assessed using the multispectral camera as described by Tan et al. (2021). Meanwhile, to uncover the impact of the *Streptomyces* strain ST03 on the expression of genes related to plant defense response, plants of each treatment were harvested at four different time points at dai 1, 2, 4, and 8 (five plants per each time-point) (Supplementary Figure 1E). Roots and leaves from each plant were extracted for RNA as described below to quantify the gene expression using RT-qPCR.

### RNA Extraction and RT-qPCR

Total RNA from roots and leaves was extracted using TRizol reagent (Sigma Aldrich, Belgium) according to the manufacturer’s instructions. RNA concentration was determined using a Quantus fluorometer (Promega, Netherlands). cDNA of each RNA template was synthesized using the iScript^TM^ kit (Bio-Rad, Belgium) and diluted five times with nuclease-free water. The quantitative reverse transcription PCR (RT-qPCR) assay was performed using a CFX96 Tough Real-time PCR Detection System (Bio-Rad, Belgium). Each PCR reaction contained 6.3 μL Gotagq^®^PCR master mix (Promega, Netherlands), 2 μL cDNA, 0.6 μL each primer (5 μM), and 0.2 μL CXR dye (Promega, Netherlands), and 2.3 μL nuclease-free water. The thermal program was set up as follows: 95°C for 3 min; 39 cycles of 95°C for 10 s, and 60°C for 30 s, followed by a melting curve acquisition from 65 to 95°C with the rate of 0.5°C s^–1^. The primers used for all target genes are shown in Supplementary Table 1. Elongation factor 1α (*EF-1*α) and β tubulin (β*-TUB*) primers were used as housekeeping genes. Gene expression analysis was done using qBase^+^ software (Biogazelle, Zwijnaarde, Belgium). Fold change was computed by diving the CNRQ values (calibrated normalized relative quantities) of the treated samples by values of the control samples. In each gene, four biological replicates and two technical replicates were done.

### Quantification of Fumonisins by LC-MS/MS

Quantification of fumonisin B_1_, B_2_, and B_3_ in each maize ear was performed using a Waters Acquity HPLC-Quattro Premier XE mass spectrometer (Waters, Milford, MA, United States) in positive electrospray ionization (ESI^+^) mode. Chromatographic separation was performed using a Symmetry C18 (150 mm × 2.1 mm, i.d. 5 μm) column with a guard column (10 mm × 2.1 mm i.d.) of the same material (Waters, Zellik, Belgium). A clean-up procedure was carried out prior to injecting samples into the device. Briefly, 100 μL de-epoxy-deoxynivalenol (DOM) (50 ng μL^–1^) were added to a Falcon tube containing 5 g of each ground sample as an internal standard. Each sample was extracted with 10 mL ethyl acetate/folic acid (99/1, v/v) by agitating for 15 h on an overhead shaker. The Falcon tubes were centrifuged at 4000 *g* for 15 min. Each upper layer was transferred into a new tube and evaporated using a nitrogen flow at 40°C, 1 atm. The pellet was re-dissolved in 200 μL injection solvent {consisting of mobile phase A [water/methanol/acetic acid (94/5/1, v/v/v) + 5 mM ammonium acetate] and mobile phase B [methanol/water/acetic acid (97/2/1, v/v/v) + 5 mM ammonium acetate] with a ratio of A/B (60/40, v/v)}; and the mixture was defatted with 200 μL hexane and subsequently filtrated using a centrifuge filter at 10000 *g* for 5 min. Finally, each filtrate was conveyed into an HPLC vial. The liquid chromatography coupled with tandem mass spectrometry (LC-MS/MS) method was described by De Boevre et al. (2012). Five biological replicates were done per treatment.

### Quantification of *Fusarium verticillioides* DNA in Soil by Quantitative PCR

A quantitative PCR (qPCR) assay was conducted in a CFX96 Touch Real-Time PCR Detection System (BIO-RAD, Temse, Belgium) as described by Tran et al. (2021a). Briefly, total genomic DNA extraction was performed from 100 mg for each soil sample, and 50 mg for pure *F. verticillioides* mycelia using the E.Z.N.A^®^ Soil DNA Kit (VWR International, Leuven, Belgium). DNA concentration was quantified with a Quantus fluorometer (Promega, Leiden, Netherlands). Each reaction mixture contained 6.3 μL Gotag^®^ qPCR Master Mix (Promega, Leiden, Netherlands), 2 μL DNA template, 0.6 μL each primer *FVer* (5 μM), 0.2 μL CXR reference dye (Promega, Leiden, Netherlands), and nuclease-free water up to a total volume of 12 μL. The PCR thermal cycling program was set-up as follows: 95°C for 3 min; 39 cycles of 95°C for 10 s, and 60°C for 30s, followed by a melting curve acquisition from 65 to 95°C. A standard curve was generated based on C_t_ (threshold cycle) values by using tenfold serial dilutions of the pure *F. verticillioides* DNA (ranging from 1 ng to 1 × 10^–5^ ng/μL). The amount of fungal DNA in samples was calculated from C_t_ values using the standard curve. Each sample calculation was performed twice. Five biological replicates were undertaken per treatment.

### Statistical Analysis

All heat maps and boxplots were generated using the R software v.4.0.2 with the packages ggplot2 and gplots^1^. The normal distribution of data was tested using the Shapiro–Wilk test. The one-way ANOVA test followed by a *post hoc* Tukey test was used in a case of the normal distribution, otherwise, a non-parametric Kruskal–Wallis test and a *post hoc* Dunn’s test were applied. As for statistical analyses in gene expression, fold change (FC) was used in the case of up-regulation, while for down-regulation log [FC] base 2 (Log_2_FC) was used. All analyses were tested at a significance level of α = 0.05.

## Results

### *In vitro* Direct Antagonism of *Streptomyces* Strains Against *Fusarium* Species

**FIGURE 1:**
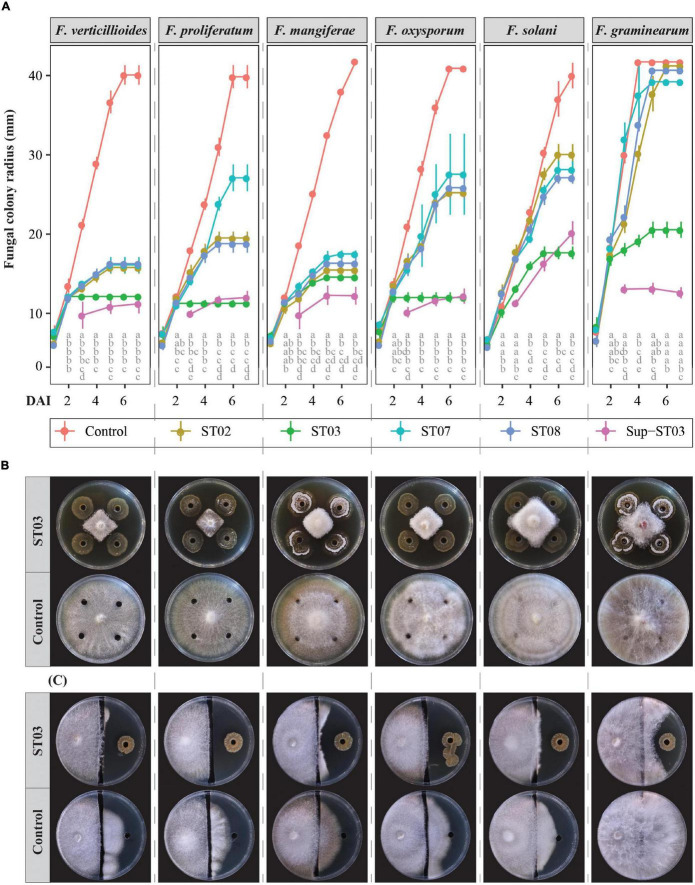
*In vitro* antagonism of four *Streptomyces* strains (ST02, ST03, ST07, and ST08) against six *Fusarium* species. **(A)** Fungal colony radii (mm) by days of inoculation (dai) between 1 and 7 dai. Four cell suspensions of ST02, ST03, ST07, and ST08, respectively, and one 10-time-concentrated supernatant of the strain ST03 (Sup-ST03) were used. Water or tryptic soy broth were used as the mock controls. Different letters by columns pinpoint a significant difference between treatments at each time point using an ANOVA test and a *post hoc* Turkey test at α = 0.05. **(B)** An *in vitro* diffusion assay of the ST03 cell suspension at 7 dai against 6 *Fusarium* species as **(A)**. **(C)** An *in vitro* volatile assay of the strain ST03 at 11 dai. Four biological replicates were done per treatment. The experiment was repeated twice.

In this study, all the rhizobacterial strains were identified as *Streptomyces* species. The *in vitro* bioassays showed an effective and stronger direct antagonism of the strain ST03 against six *Fusarium* pathogens compared to the other rhizobacterial strains (Figure 1A). It is clear that in the *in vitro* diffusion assay, an early and significantly inhibitory indication was observed against *F. verticillioides*, *F. proliferatum*, *F. oxysporum*, and *F. graminearum* when co-applied with the strain ST03 at 2 dai and at 3 dai for *F. mangiferae* and *F. solani* in comparison to the mock control. Surprisingly, *F. verticillioides*, *F. proliferatum*, and *F. oxysporum* were thoroughly inhibited by the strain ST03 from 2 dai onward since their colony radii remained unchanged between 2 and 7 dai. It was coherent when its inhibition efficacy against these three pathogens was highest and significant at 7 dai compared to the other bacterial strains (Figure 1B and Supplementary Figure 2). The results also indicated absolute but a later inhibition of *F. mangiferae*, *F. solani*, and *F. graminearum* when co-inoculated with the strain ST03 at 5 dai (Figure 1A), which explained lower inhibition efficacy at 7 dai, of 65 ± 1%, 56 ± 2%, and 51 ± 2%, respectively (Figure 1B and Supplementary Figure 2). It was significantly higher than the inhibition efficacy of the other strains ST02, ST07, and ST08. An obvious example is that at 7 dai the inhibition efficacy against *F. graminearum* by the strain ST03 was 51 ± 2%, while this was not significantly effective for the other ones, for example, ST02 (3 ± 2%, *p* < 0.001), ST07 (6 ± 8%, *p* < 0.001), and ST08 (4 ± 2%, *p* < 0.001) (Supplementary Figure 2). Remarkably, the antagonistic efficacy of the strain ST03 reached above 50% at 5 dai for all the pathogens except for *F. solani* (Supplementary Figure 2). We, therefore, used this strain ST03 for in-depth studies.

To assess the impact of volatile compounds produced by strain ST03, we used an *in vitro* volatile bioassay. Even though this assay did not indicate the role of volatile metabolites for the antagonism against the pathogens, it strengthened the diffusion-based antagonism since the inhibition zones were more transparent for each pathogen compared to the mock control (Figure 1C). At 11 dai, *F. verticillioides*, *F. proliferatum*, and *F. oxysporum* did not grow onto the other side inoculated with strain ST03, whereas for the mock control plates the fungi colonized the other side from the channel by approx. 25 mm. As similar to the diffusion assay, the inhibition efficacy was less for *F. mangiferae*, *F. solani*, and especially for *F. graminearum* (Figure 1C).

**FIGURE 2:**
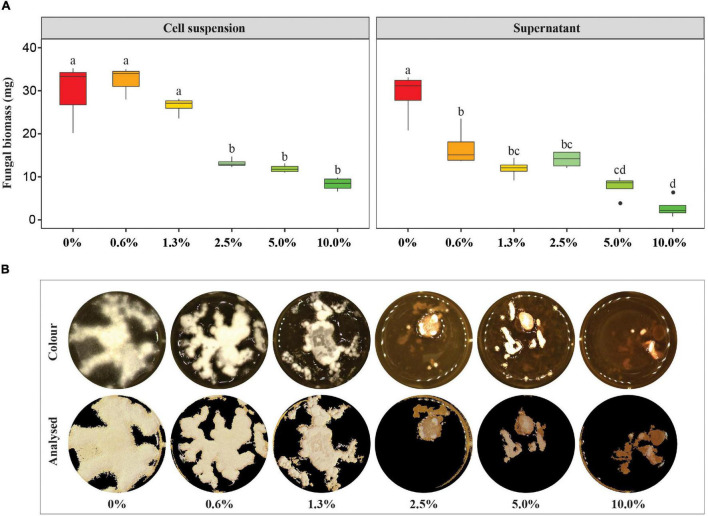
Impact of the *Streptomyces* strain ST03 on the production of biomass of *Fusarium verticillioides* in liquid medium. **(A)** Dried *F. verticillioides* biomass (g) by treatments. An initial concentration of the cell suspension was 5×10^7^ CFU mL^– 1^, the supernatant was concentrated 10 times before application. Different letters above each box depict the significant difference between treatments using an ANOVA test and a *post hoc* Turkey test at a significant level of α = 0.05. A mock control (0% treatment) was inoculated with tryptic soy broth (TSB). **(B)** Growth surface of *F. verticillioides* in tryptic soy broth supplemented with the supernatant at different concentrations ranging 0 – 10% at 7 days of inoculation. Four biological replicates were done per treatment. The experiment was repeated twice.

The high antagonistic efficacy of strain ST03 was proven in the other *in vitro* assay using a liquid medium. It was confirmed by a significant decrease in biomass production by *F. verticillioides* when co-inoculated with the cell suspension of strain ST03, ranging from 2.5 to 10% (Figure 2A). The fungal growth was remarkably suppressed when co-applied with 2.5% of strain ST03 equivalently with 1.25 × 10^6^ CFU mL^–1^ because there was a significant reduction of dried biomass by 59% compared to the mock control (0% treatment), specifically 13 ± 1 and 32 ± 9 mg, respectively (*p* = 0.021). This trend also occurred in the treatments co-inoculated with 5% (12 ± 1 mg, *p* = 0.017) and 10% (8 ± 2 mg, *p* = 0.005) of the cell suspension, but the change was not different from the treatment 2.5% (Figure 2A).

We hypothesized whether extracellular compounds secreted by strain ST03 in the supernatant are involved in the interaction with the pathogens. To explore this, we concentrated the supernatant 10 times after separating from the cell and applied both *in vitro* bioassays in the same way with the cell suspension. As indicated in the plate diffusion assay (Figure 1A), the supernatant of strain ST03 had a significantly effective antagonism against six *Fusarium* species. It was clear that the growth of all fungi were strongly inhibited when co-applied with the supernatant at 3 dai by their smaller radii compared to the mock controls. For example, the colony radius for *F. verticillioides* was 10 ± 2 cm vs. 21 ± 1 cm (*p* < 0.001), *F. proliferatum* (10 ± 0.5 cm vs. 18 ± 0.3 cm, *p* < 0.001), and *F. graminearum* (13 ± 1 cm vs. 30 ± 1 cm, *p* < 0.001). Surprisingly, an absolute inhibition was observed at 3 dai for all the pathogens with the exclusion of *F. solani* when the fungi did not grow further after 3 days of being inoculated with the supernatant (Figure 1A). This phenomenon resulted in a high inhibition efficacy against these pathogens of over 60% at 5 dai and over 70% at 7 dai (Supplementary Figure 2).

In addition, the antifungal activity of this supernatant was demonstrated in the liquid assay (Figure 2). The fungus *F. verticillioides* was significantly inhibited when co-applied with 0.6% of the supernatant at 7 dai by a significant decrease in dried biomass compared to the mock control (0%), given 17 ± 5 mg vs. 29 ± 6 mg, respectively (*p* = 0.016) (Figure 2A). Although no significant change in the biomass production of the fungus in a range of between 0.6 and 2.5% was observed, more suppression was observed at 5% (8 ± 3 mg dried biomass, *p* < 0.010), and 10% (3 ± 2 mg dried biomass, *p* < 0.001) than 2.5% (14 ± 2 mg) (Figure 2A). Moreover, it was clear that the growth surface of the fungus was remarkably smaller in the treatments 2.5, 5, and 10% compared to the mock control (Figure 2B). Moreover, an inhibition efficacy calculated at a concentration of 10% was 90 ± 8%.

In conclusion, the *in vitro* antagonistic bioassays show that of the four *Streptomyces* strains, strain ST03 had the highest inhibition efficacy against all the surveyed *Fusarium* pathogens. The data also indicate the antifungal potential of the extracellular compounds in the supernatant produced by this strain.

### *In vivo* Direct Antagonism of *Streptomyces* Strain ST03 Against *Fusarium verticillioides*

To confirm the antagonistic efficacy of strain ST03 from the *in vitro* parts, we performed two *in vivo* bioassays on maize cobs and maize seedlings infected with *F. verticillioides*. We focused on *F. verticillioides* since it is the main causal agent of FER and predominantly occurs in maize fields worldwide.

**FIGURE 3:**
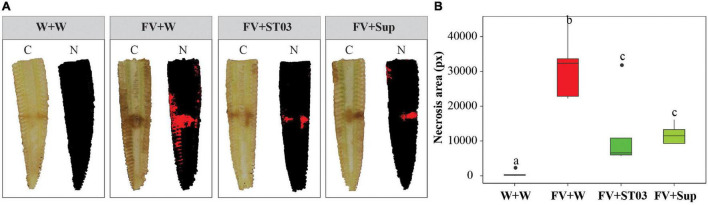
Impact of the *Streptomyces* strain ST03 when co-applied with *Fusarium verticillioides* on maize cobs. **(A)** Imaging inside maize cobs by treatments at 7 days using a multispectral camera. Color images **(C)**, and necrosis images in red color (N). **(B)** Necrosis area (px) by treatments. Negative maize cob control was inoculated with sterile water (W + W), positive maize cob control comprised *F. verticillioides* and sterile water (FV + W), two biocontrol treatments included *F. verticillioides* and the *Streptomyces* cell suspension (FV + ST03)/or the supernatant of the strain ST03 (FV + Sup). Different letters above each box depict the significant difference between treatments using a Kruskal–Wallis test followed by a *post hoc* Dunn’s test at a significant level of α = 0.05. Five biological replicates were performed per treatment. The experiment was repeated twice.

First, we assessed the impact of strain ST03 when co-applied with *F. verticillioides* on maize cobs (Figure 3). Data illustrated that the infection of the fungus to maize cobs was significantly counteracted when co-inoculated with the cell suspension or supernatant of strain ST03 (Figure 3). By using the multispectral camera, a high infection level of *F. verticillioides* on maize cobs (red area) (FV + W) was observed with a larger lesion area when compared to the negative control (W + W) (p = 0.001) (Figures 3A,B). However, it significantly reduced when co-applied with the cell suspension (FV + ST03) or supernatant (FV + Sup) of strain ST03 by smaller surface areas (*p* = 0.008), respectively (Figure 3B).

**FIGURE 4:**
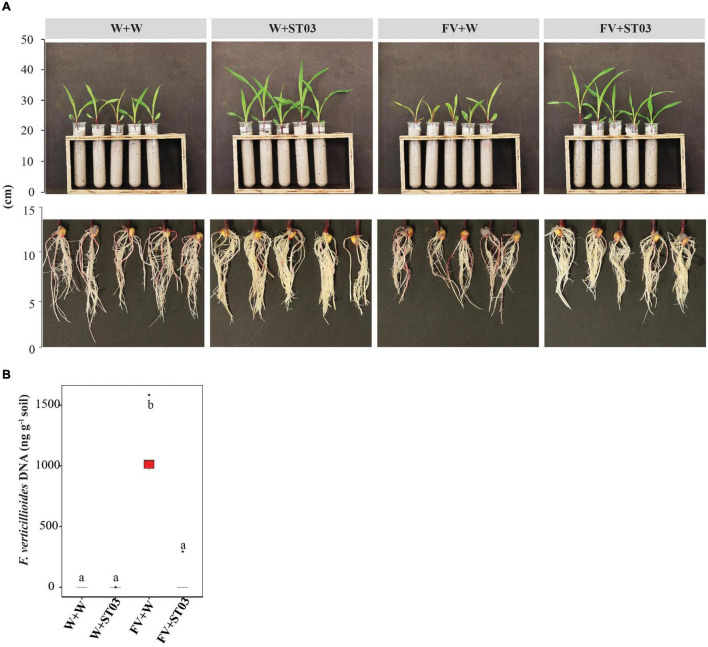
*In vivo* direct antagonism of the *Streptomyces* strain ST03 on maize seedlings based on levels of *Fusarium verticillioides* DNA in soil. **(A)** Plants and roots at 14 days after inoculation. **(B)** The fungal DNA content (ng g^– 1^) in soil by treatments using a qPCR method. *F. verticillioides*-infected maize seeds were planted in soil pre-inoculated with the biocontrol strain ST03 (FV + ST03) and planted in soil mock-inoculated with sterile water (FV + W); *F. verticillioides* non-infected maize seeds were planted in soil pre-inoculated with the biocontrol strain (W + ST03) and planted in mock soil (W + W). A hybrid CP888 maize line was used. Different letters above each box depict the significant difference between treatments using a Kruskal–Wallis test followed by a *post hoc* Dunn’s test at a significant level of α = 0.05. Five biological replicates were performed per treatment. The experiment was repeated twice.

For the *in vivo* bioassay on maize seedlings, we checked the direct antagonism of the biocontrol strain ST03 by quantification of the *F. verticillioides* DNA in soil surrounding roots upon 14 days of infection. We planted *F. verticillioides*-infected CP888 seeds into two types of soil with and without pre-inoculation with the biocontrol strain ST03 (Figure 4A). qPCR data indicate that a higher level of *F. verticillioides* DNA was present in soil without pre-inoculation with strain ST03 (FV + W), compared to soil that was pre-inoculated (*p* = 0.009) (Figure 4B). This was consistent with the phenotypic characteristics of roots and lower levels of infection were observed in plants treated with the biocontrol strain compared to the non-treated plants (Figure 4A). All evidence shows that strain ST03 could suppress *F. verticillioides* through a direct antagonism upon infection on maize seedlings.

To sum up, both *in vivo* bioassays on maize cobs and plants demonstrate the direct antagonism of the biocontrol strain ST03 against *F. verticillioides*. In addition to cell suspension, the extracellular compounds in the supernatant secreted by this strain also show a strong inhibition against *F. verticillioides*.

### Impact of *Streptomyces* Strain ST03 on Fumonisin Production When Co-applied With *Fusarium verticillioides* on Maize Cobs

**FIGURE 5:**
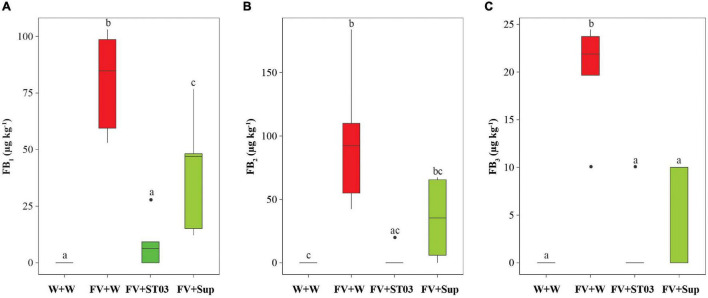
Impact of the *Streptomyces* strain ST03 on the production of fumonisin B_1_ (FB_1_) **(A)**, FB_2_ **(B)**, and FB_3_ **(C)** by *Fusarium verticillioides* on maize cobs. The negative maize cob control was inoculated with sterile water (W + W), positive maize cob control comprised *F. verticillioides* and sterile water (FV + W), the two biocontrol treatments included *F. verticillioides* and the *Streptomyces* cell suspension (FV + ST03) or the supernatant of the strain ST03 (FV + Sup). Different letters above each box depict the significant difference using a Kruskal–Wallis test followed by a *post hoc* Dunn’s test at a significant level of α = 0.05. Five biological replicates were performed per treatment. The experiment was repeated twice.

The *in vivo* antagonistic bioassay on maize cobs indicated the inhibition efficacy of both cell suspension and supernatant of the strain ST03 by the reduction of the necrosis area (Figure 3). To evaluate the impact on fumonisin production by *F. verticillioides* we measured FBs in these cobs. Data show that levels of FB_1_, FB_2_, and FB_3_ in maize cobs treated with the cell suspension (FV + ST03) or the supernatant (FV + Sup) was significantly lower than in the non-treated maize cobs (FV + W) (Figure 5). A significantly lower FB_1_ median level was found in maize cobs treated with the cell suspension compared to the non-treated maize cobs, given 9 ± 5 μg kg^–1^ and 80 ± 10 μg kg^–1^, respectively (*p* = 0.012). A similar result was observed for FB_2_, amounting 0 ± 4 μg kg^–1^ (in FV + ST03 treatment) and 92 ± 25 μg kg^–1^ (in FV + W treatment) (*p* = 0.009), and for FB_3_ (0 ± 2 μg kg^–1^ vs. 22 ± 3 μg kg^–1^, *p* = 0.013). Similar results were also observed for the maize cobs treated with the supernatant (FV + Sup) except for FB_2_ (Figure 5). Collectively, the biocontrol strain was capable of suppressing the production of fumonisins by *F. verticillioides*.

### Impact of *Streptomyces* Strain ST03 on Plant Growth When Co-applied With *Fusarium verticillioides* on Maize Seedlings

**FIGURE 6:**
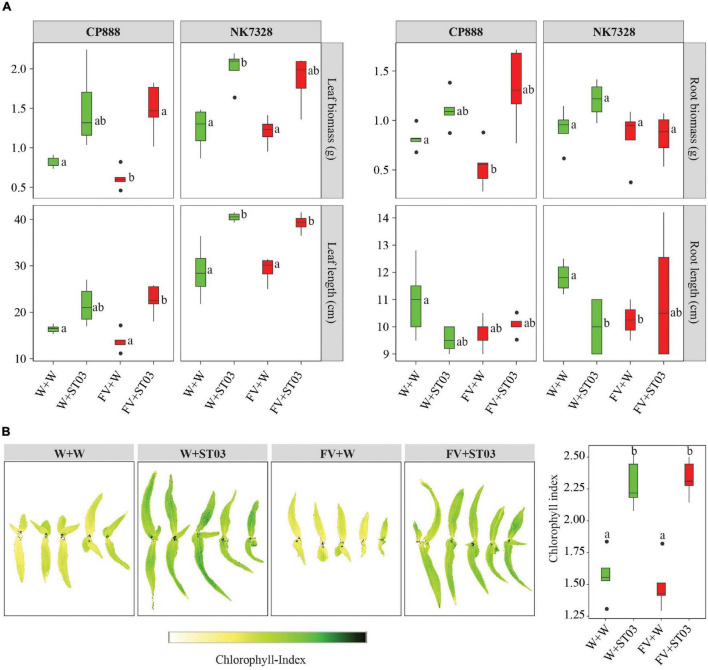
**(A)** Impact of the *Streptomyces* strain ST03 on plant growth using two hybrid maize cultivars CP888 and *Bt/GT* NK7328 at 14 days after planting. **(B)** Chlorophyll level by treatments on CP888 plants. Chlorophyll content was assessed using a multispectral camera; white is a low chlorophyll index (ChlIdx) and dark green is high. *F. verticillioides*-infected maize seeds were planted in soil pre-inoculated with the biocontrol strain ST03 (FV + ST03) and planted in mock-inoculated with sterile water soil (FV + W); *F. verticillioides* non-infected maize seeds were planted in soil pre-inoculated with the biocontrol strain (W + ST03), or planted in mock soil (W + W). Different letters above each box depict the significant difference between treatments by cultivars using a Kruskal–Wallis test followed by a *post hoc* Dunn’s test at a significant level of α = 0.05. Five biological replicates were done per treatment. The experiment was repeated twice.

After uncovering the direct antagonism of the biocontrol strain on maize seedlings by the reduction of the DNA level of *F. verticillioides* in soil (Figure 4), we explored whether the growth of plants was impacted. To examine this, we used two hybrid maize lines CP888 and *Bt/GT* NK7328 predominantly planted in the central highlands of Vietnam (Tran et al., 2021c). For line CP888, the growth of plants infected with the fungus (FV + W) was negatively affected with significantly lower fresh leaf and root biomass weight when compared to the control non-infected plants (Figure 6A). Moreover, a reduction of chlorophyll index was observed in the infected plants versus the control plants (Figure 6B). For line *Bt/GT* NK7328, although the plants were less infected compared to line CP888, their root length was also significantly shorter than the control NK7328 plants, of 10.3 ± 0.3 and 11.8 ± 0.3 cm, respectively (*p* = 0.029) (Figure 6A).

However, when co-applied with the biocontrol strain ST03 (FV + ST03), the growth of plants was significantly stimulated compared to the infected and the control plants of both maize lines. For example, for line CP888, the leaf length of plants co-applied with strain ST03 was 22.5 ± 1.4 cm (FV + ST03) versus 13.0 ± 1.0 cm of the infected plants (FV + W) (*p* = 0.029). Likewise, for *Bt/GT* NK7328 plants the length was 39.4 ± 1.0 cm (FV + ST03) vs. 30.2 ± 3.1 cm (FV + W) (*p* = 0.029) (Figure 6A). In both cultivars, the growth of the plants when co-applied with the biocontrol strain was also better than the growth of the control plants (W + W). It was evidenced by higher fresh biomass and leaf length (Figure 6A). Chlorophyll index was higher in the leaves of CP888 plants when co-applied with strain ST03, given 2.3 ± 0.1 (FV + ST03) vs. 1.4 ± 0.1 (FV + W) (*p* = 8e-4) or vs. 1.6 ± 0.1 (W + W) (*p* = 0.008) (Figure 6B).

We hypothesized whether this biocontrol strain ST03 acts as a plant growth-promotion agent on top of its direct antagonism. To investigate this, we examined growth indicators of plants that were applied with strain ST03 alone (W + ST03). Data revealed that the plant growth of both lines was significantly promoted with the presence of ST03 strain compared to the control plants (W + W). For example, in *Bt/GT* NK7328 line, leaf length was longer at 40.1 ± 0.5 (W + ST03) vs. 28.4 ± 3.1 cm (W + W) (*p* = 0.029), and a higher leaf biomass of 2.1 ± 0.1 vs. 1.3 ± 0.1 g (*p* = 0.029) (Figure 6A). Similar results were observed in plants of line CP888 (Figure 6A). The chlorophyll index of plants applied with strain ST03 was significantly higher than that of the control plants, given 2.2 ± 0.1 and 1.6 ± 0.1, respectively (*p* = 0.008) (Figure 6B). Nevertheless, the primary root length of plants applied with the biocontrol strain was shorter compared to the control. It was true in both lines CP888 [of 9.5 ± 0.2 (W + ST03) vs. 11 ± 0.6 (W + W), (*p* = 0.145) and *Bt/GT* NK7328 (of 10.0 ± 0.6 vs. 11.8 ± 0.3, (*p* = 0.028)]. Higher fresh root biomass was observed in plants applied with strain ST03 compared to the control plants (Figure 6A) since root hair formation was induced as shown in Figure 4A.

From the aforementioned data, we can conclude that the *Streptomyces* strain ST03 not only hampered the growth of *F. verticillioides* but also stimulated the growth of plants.

### Impact of *Streptomyces* Strain ST03 on Intrinsic Plant Defense Response and Plant Growth Promoting Hormones When Co-applied With *Fusarium verticillioides* on Maize Seedlings

Concerning the action of this biocontrol strain on modulating plant growth promoting hormones, the involvement of genes associated with the metabolic pathways of auxin/indole-3-acetic acid (Auxin/IAA), abscisic acid (ABA), and gibberellic acid (GA) were examined. In the literature, the timely activation of key genes in the biosynthesis pathways of salicylic acid (SA), jasmonic acid (JA), 1,4-benzoxazine-3-ones (BXs), and pathogenesis-related proteins (PRs) plays a dominant role in maize resistance to fungal plant pathogens (Bacon et al., 2007; Ding et al., 2015; Wang et al., 2016). Thus, we further uncovered modes of action of the biocontrol strain ST03 on these secondary metabolism-related genes.

#### The Action on Key Genes in the Biosynthesis of Auxin, Gibberellic Acid, and Abscisic Acid

To assess the impacts of the biocontrol strain on the biosynthesis of the typical plant growth hormones Auxin/IAA, GA, and ABA, we verified the expression profiles of the genes encoding the auxin responsive factors 1 and 2 (*ARF1* and *ARF2)*, the auxin transporter-like protein (*AUX1)* (for auxin signaling pathway), the glycine-rich protein (*ABI)* (for ABA signaling pathway), and the anther ear 1 (*AN1)* (for GA signaling pathway). These genes were selected because of their important biological function in the biosynthesis pathways of these phytohormones. For example, *ARF1* and *ARF2* encode two auxin transcription factors, while *AUX1* encodes an auxin influx transporter (Zhang et al., 2019). Gene *ABI* plays a regulatory role in ABA signaling by encoding a glycine-rich protein (Ding et al., 2015). Finally, *AN1* is capable of regulating the GA phytohormone biosynthesis in maize (Zhang et al., 2019).

**FIGURE 7:**
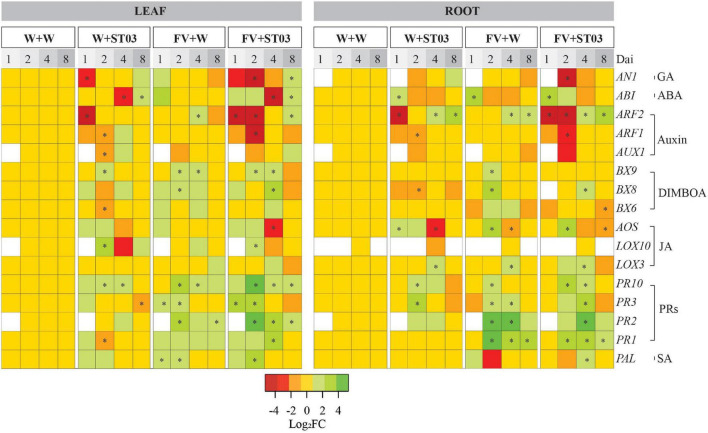
Heat map of the differential gene expression levels in leaf and root by treatments at four-time points. This work applied *Bt/GT* NK7328-maize line. The colors indicate the FC changes (FC) of gene expression in log base 2. Up-regulation of each gene is shown in green (increased abundance), down-regulation is shown in red (decreased abundance), no change is shown in yellow, and absence of expression is indicated in white. Salicylic acid (SA); jasmonic acid (JA); 2,4-dihydroxy-7-methoxy-1,4-benzoxazin-3-one (DIMBOA); abscisic acid (ABA); gibberellic acid (GA); auxin/indole-3-acetic acid (Aux/IAA). dai, days after inoculation. Defense response was monitored at 1, 2, 4, and 8 dai. *F. verticillioides*-infected maize seeds were planted in soil pre-inoculated with the biocontrol strain ST03 (FV + ST03) and planted in mock-inoculated with sterile water soil (FV + W); *F. verticillioides* non-infected maize seeds were planted in soil pre-inoculated with the biocontrol strain (W + ST03); or planted in mock soil (W + W). The final concentration of *Streptomyces* ST03 in soil was 10^6^ CFU g^– 1^. At each time point, four biological replicates were done per treatment. The experiment was repeated twice. Symbol (*) depicts the significant difference between treatments and the control mock plants using a one-tailed Kruskal–Wallis test at a significant level of α = 0.05.

In roots, at early time points 1 and 2 dai, a clear downregulation of *AUX1*, *ARF1*, and *ARF2* was observed in plants applied with strain ST03 only (W + ST03 treatment) (Figure 7). However, later at 4 and 8 dai, *ARF2* was significantly up-regulated, respectively representing 2.7-fold (*p* = 0.029) and 5.5-fold (*p* = 0.029), while *ARF1* and *AUX1* returned to the basal levels compared to the control plants. Similar trends were observed when co-applied with the pathogen (FV + ST03 treatment). Especially, at 2 dai, there was a concomitant downregulation of *AUX1* (Log_2_FC = –2.7, one-tailed *p* = 0.065), *ARF1* (Log_2_FC = –3.4, *p* = 0.029), and *ARF2* (Log_2_FC = –9.7, *p* = 0.029). By contrast, when applied with *F. verticillioides* alone, at 1 and 2 dai, the expression levels of *AUX1*, *ARF1*, and *ARF2* were not different from the control plants. Comparable to the other treatments, *ARF2* was upregulated at 4 (2.7-fold, *p* = 0.029) and 8 (3.9-fold, *p* = 0.029) dai. Interestingly, similar results were observed in leaves in all the treatments.

For the GA biosynthesis-related gene *AN1*, in roots, at 2 dai, a very small downregulation was observed at this gene [Log_2_FC = –1, *p* = 0.1] when applied with the biocontrol strain (Figure 7). Later *AN1* was induced at 8 dai (4.7-fold, *p* = 0.029). In the concomitant presence of *F. verticillioides* and the biocontrol strain, a strong suppression of *AN1* was observed at 2 dai (Log_2_FC = –5.7, one-tailed *p* = 0.065), subsequently, at 8 dai, this gene returned to the basal level as in the control plants. When applied with *F. verticillioides* alone, the expression level of *AN1* was not different from the control plants at all time points. In leaves, similar trends of *AN1* expression were obtained from roots for all treatments after 1 dai.

For the profile of ABA-related gene *ABI* in roots when co-applied with the pathogen (FV + ST03), at the earlier time-points 1 and 2 dai *ABI* was triggered 8.8-fold (*p* = 0.029) and 2.2-fold (one-tailed *p* = 0.057) respectively and returned to the basal level. A similar trend was observed at the singular inoculation of *F. verticillioides*. Similar observations were obtained in leaves by treatments.

In conclusion, the findings indicate that the preinoculation of biocontrol strain ST03 in the soil resulted in a downregulation of the auxin responsive genes *ARF1*, *ARF2*, and *AUX1*, and the GA-related gene *AN1* at earlier time-points even in absence of *F. verticillioides*. Afterward, the expression of these genes was similar to the control treatment or upregulated from 4 dai onward, particularly, *ARF2*. By contrast, the singular infection of *F. verticillioides* did not impact the expression levels of these genes.

#### The Action on Key Genes in the Biosynthesis of Salicylic Acid, Jasmonic Acid, and Pathogenesis-Related Proteins

To assess the action on the SA, JA, and PRs biosynthesis, we used genes encoding the hallmark enzymes phenylalanine ammonialyase (*PAL)* (for SA pathway), allene oxide synthase (*AOS)*, and lipoxygenases (*LOX3* and *LOX10)* (for JA pathway), and PR proteins (*PR1*, *PR2*, *PR3*, and *PR10)* (for PRs biosynthesis). The activation of these genes has been demonstrated to be involved in the defense responses of maize to biotic stresses (Lanubile et al., 2010; Tzin et al., 2017; Zhang et al., 2019).

In roots, for plants applied with train ST03 alone (W + ST03), the expression level of *PAL* remained unchanged compared to the mock control plants at all time points (Figure 7). Similar results were observed in plants co-applied with the pathogen (FV + ST03) with the exception at 4 dai where a small but significant induction of *PAL* (3.3-fold, *p* = 0.028) was observed. In leaves, at early time-points 1 and 2 dai, *PAL* seemed to be upregulated in all the treatments (Figure 7). Notably, at 2 dai, the gene expression levels were high of respectively, 5.4-fold (in W + ST03, one-tailed *p* = 0.15), 13.4-fold (in FV + ST03, *p* = 0.029), and 4.7-fold (in FV + W, *p* = 0.029).

Turning to the effects on *PR1*, *PR2*, *PR3*, and *PR10* genes, in roots, at 2 dai, the singular presence of the biocontrol strain resulted in a strong induction of *PR2* (6.1-fold, *p* = 0.028), *PR3* (6.5-fold, *p* = 0.028), and *PR10* (5.2-fold, *p* = 0.028) when compared to the mock control plants (Figure 7). The expression levels of *PR2* and *PR10* subsequently reduced till the basal levels at 4 dai onward. Remarkably, the concomitant application of *F. verticillioides* and the biocontrol strain ST03 resulted in a strong induction of the four PRs encoding genes at 2 and 4 dai. For example, at 4 dai, the expression levels were 6.3-fold of *PR1* (*p* = 0.028), 108.2-fold of *PR2* (*p* = 0.029), 11.3-fold of *PR3* (*p* = 0.029), and 3.6-fold of *PR10* (*p* = 0.029) compared to the mock control treatment (Figure 7). Similar results were observed for plants applied with the pathogen alone, indicating that *F. verticillioides* triggers the PR genes.

In leaves, when applied with the biocontrol strain alone, the expression of *PR1*, *PR2*, *PR3*, and *PR10* was observed at the basal levels as in the control plants. When in co-inoculation of *F. verticillioides* and the biocontrol strain, at 1 dai, a small induction of *PR1*, *PR3*, and *PR10* was present, particularly, there was a significantly clear upregulation of *PR3* (8.8-fold, *p* = 0.029) compared to the mock control treatment. Remarkably, at 2 dai, and 4 dai, these genes were still upregulated together with the concomitant upregulation of the *PR2* gene. Later, at 8 dai, all the PRs genes were regulated toward the basal levels as in the control treatment. Similar trends were observed when applied with the pathogen alone (Figure 7). Nonetheless, at 2 dai, data show that the expression levels of *PR2*, *PR3*, and *PR10* was lower in plants applied with the pathogen than in plants co-applied with the biocontrol strain and *F. verticillioides*, representing *PR2* [8.1-fold (FV + W) vs. 83.8-fold (FV + ST03)], *PR3* (3.7-fold vs. 17-fold), and *PR10* (7-fold vs. 37-fold) (Figure 7).

As for the expression of JA pathway-related genes, in roots, at 1 and 2 dai, a very small induction of *AOS*, but not of *LOX3* and *LOX10* was observed when applied with the biocontrol strain alone (Figure 7). Then, at 4 dai, *AOS* was suppressed with Log_2_FC levels of respectively, –2.8 (*p* = 0.029), while *LOX3* showed a small upregulation (4.4-fold, *p* = 0.029). When co-applied with the pathogen (FV + ST03), at 2 dai, an upregulation of *AOS* (5.6-fold, *p* = 0.029) and *LOX3* (1.9-fold, *p* = 0.029) was observed. *LOX3* was upregulated (3.4-fold, *p* = 0.029) at 4 dai, and then was downregulated at 8 dai (Log_2_FC = –1.8, *p* = 0.029). Similar trends were observed in plants applied with the pathogen alone (Figure 7).

In leaves, at 1 dai, only *AOS* was slightly induced by the biocontrol strain (3.1-fold, one-tailed *p* = 0.25) (Figure 7). Subsequently, the small induction of *AOS* (2-fold, one-tailed *p* = 0.15) continued to be observed at 2 dai besides induction of *LOX10* (5.8-fold, *p* = 0.029). At 4 dai, both *AOS* and *LOX10* were down-regulated with Log_2_FC values of –1.4 (one-tailed *p* = 0.06) and –2.3 (one-tailed *p* = 0.05), respectively. At 8 dai, they were modulated back to the basal levels as in the control treatment. Similar trends were observed in plants co-applied with *F. verticillioides*. In contrast, in presence of *F. verticillioides* alone, the expression levels of *AOS*, *LOX3*, and *LOX10* were not different from the control treatment.

To sum up, the data show that the biocontrol strain ST03 slightly induced *PAL* (SA pathway), *AOS* and *LOX3* (JA pathway), and the PRs-related genes (*PR1*, *PR2*, *PR3*, and *PR10*) even in absence of a pathogen. Secondly, root infection by *F. verticillioides* resulted in a strong induction of PRs-related genes in both roots and leaves.

#### The Action on Key Genes in the Biosynthesis of 1,4-Benzoxazine-3-Ones

To determine the effect of the biocontrol strain ST03 on the biosynthesis of BXs defensive secondary metabolites, we examined three key BXs responsive genes *BX6*, *BX8*, and *BX9* in the leaves and roots of plants. The induction of these genes might increase the resistance of maize plants against *F. verticillioides*.

In roots, the expression of BXs-related genes remained unchanged in plants applied with the biocontrol strain alone (W + ST03) (Figure 7) except for a slight down-regulation of the *BX8* at 2 dai (Log_2_FC = –1.8, *p* = 0.029) compared to the mock control plants. A similar expression was observed in plants that were co-applied with the pathogen (FV + ST03). By contrast, at 2 dai, these genes were found at upregulated expression levels in plants applied with the pathogen alone (FV + W), especially for *BX8* with the 5.5-fold change (*p* = 0.029), demonstrating that *F. verticillioides* could induce the BXs-mediated resistance response of maize roots (Figure 7).

Considering the responses of leaves, we observed a small but non-significant regulation of the BXs-related genes in plants when applied with the *Streptomyces* strain only compared to the mock control plants. On the contrary, a slight induction of these BXs-related genes appeared in the leaves of the plants when co-applied with the biocontrol strain and *F. verticillioides*. Specifically, at 2 dai, the expression level of *BX9* significantly increased (2.8-fold, *p* = 0.029), and notably, a concomitant upregulation of *BX6*, *BX8*, and *BX9* was present at 4 dai, of which *BX8* showed a significant expression level with 6.5-fold change (*p* = 0.029) compared to the mock control plants (Figure 7). At 2 dai the BXs-related genes *BX8* (4.7-fold, *p* = 0.029) and BX9 (2.5-fold, *p* = 0.029) were slightly triggered upon infection of *F. verticillioides*.

In conclusion, the *BX6*, *BX8*, and *BX9* expression data show that the biocontrol strain ST03 did not induce these genes in both leaves and roots in the absence of the pathogen. While a small induction was observed in leaves when in concomitant presence of *F. verticillioides*. The BXs pathway in maize seedlings was also slightly triggered by a singular *F. verticillioides* infection.

## Discussion

*Fusarium* ear rot is a severe disease in maize mainly caused by *F. verticillioides*, which not only reduces crop production and grain quality but also poses a health risk due to the production of fumonisins, which are possibly carcinogenic to humans. This fungal phytopathogen increasingly occurs in maize fields worldwide. To the best of our knowledge, no FER-resistant maize inbred lines are registered on market. Together with the strict legislation of agrochemicals, searching for rhizobacteria-based BCAs is a sustainable and eco-friendly strategy. Prior reports have documented that BCAs mainly suppress fungal crop pathogens through direct antagonism and indirect induction of the plant systemic resistance (Pineda et al., 2010; Khan et al., 2020; Raymaekers et al., 2020). Yet, to date, molecular insights into the intricate interaction of *F. verticillioides – Streptomyces* – maize are scarce.

This study demonstrated that the *Streptomyces* strain ST03 not only directly antagonizes the pathogen but also interferes with plant defense systems and promotes the growth of plants. Moreover, the *in vitro* data show a wide spectrum of direct antagonism of this biocontrol strain ST03 to other fungal crop diseases, e.g., FHB in wheat caused by *F. graminearum*, *Fusarium* basal rot (FBR) in onion caused by *F. oxysporum*.

In agreement with prior studies *Streptomyces* spp. can produce antibiosis-mediated compounds, e.g., fungal cell wall-degrading enzymes (such as 1,3-β-glucanase, and chitinase) (Getha and Vikineswary, 2002), volatile compounds (Jones and Elliot, 2017), and other toxic extracellular metabolites that can defeat the pathogen in a distance due to their rapid diffusion (Getha and Vikineswary, 2002; Faheem et al., 2015). The cell-free supernatant – a reservoir of extracellular compounds excreted by the strain ST03, showed high efficacy against the pathogens. The cell-free supernatant inhibited the proliferation of *F. verticillioides* in both *in vitro*, and *in vivo* bioassays.

Apart from the direct antagonism, the strain ST03 hampered the mycotoxin production of *F. verticillioides*. The levels of FB_1_, FB_2_, and FB_3_ were significantly reduced in the *F. verticillioides* infected cobs treated with the *Streptomyces* ST03 cell suspension or cell-free supernatant compared to the non-treated cobs. A possibility for the reduction of fumonisins is the inhibition of fungal growth by strain ST03. In agreement with Nguyen et al. (2020), *Streptomyces* spp. not only inhibit the growth of *F. verticillioides*, but also reduce fumonisin production via interfering with the fungal metabolic pathways, particularly mycotoxigenesis. Notwithstanding, the underlying mechanisms on the actions on mycotoxigenesis of *F. verticillioides* by *Streptomyces* spp. are often lacking. A study by Strub et al. (2019) has indicated that *Streptomyces* sp. suppresses the fumonisin biosynthesis-involved genes when co-cultured with *F. verticillioides.* In addition, although there is no report on biodegradation of fumonisins by *Streptomyces* spp., biodegradation of aflatoxin B_1_ by this microbial community has been reported (Harkai et al., 2016). Several studies have shown an effective biocontrol potential of *Streptomyces* spp. in suppression of the growth of *F. graminearum*, *Aspergillus flavus*, and their mycotoxin production (Boukaew et al., 2020; Colombo et al., 2020).

Interestingly, the biocontrol strain ST03 is also a plant growth-promoting candidate. Data show that the physiological and developmental index, e.g., leaf length, leaf biomass, and chlorophyll content were significantly stimulated when applied with the biocontrol strain alone or when co-applied with the pathogen. Numerous studies have demonstrated that actinobacteria can produce plant growth hormones, especially indole-3-acetic acid (IAA) (Goudjal et al., 2013; Lin and Xu, 2013). We hypothesize that the production of IAA by *Streptomyces* plays a pivotal role in the rhizosphere colonization and plant growth promotion of plants. IAA is well-known as an important signaling molecule in plant-microbe interaction (Spaepen and Vanderleyden, 2011). In the present work, it is possible that IAA produced by the *Streptomyces* strain ST03 resulted in the suppression of auxin pathway-related genes *ARF1*, *ARF2*, and *AUX1* in plants (Graphical Abstract), which could not only be a strategy used by ST03 to circumvent the plant defense system, contributing to better bacteria-plant-root interaction (Spaepen and Vanderleyden, 2011) but especially this suppression might also decrease susceptibility of plants to the pathogen (Navarro et al., 2006). A report by Ding et al. (2008) showed that repression of auxin/IAA signaling enhances the resistance of rice to *Xanthomonas oryzae* pv. *Oryzae.* This is because the IAA is an elicitor of the biosynthesis of expansins that loosen the plant cell wall, but this loosening can facilitate root penetration of the pathogen (Ding et al., 2008). Thus, inhibition of expansins activity is a result of the repression of auxin signaling.

In addition to the suppression of auxin signaling, the *AN1* expression data show that the biocontrol strain suppresses the GA biosynthesis pathway. A possibility is that *Streptomyces* strain ST03 is also capable of producing GA beside IAA, resulting in suppression of GA signaling in plants. Toumatia et al. (2016) reported that a *Streptomyces* strain has the ability to produce both IAA and GA, which promote the growth of wheat seedlings. Even though the roles of bacterial GA in plant-microbe communication are not fully understood yet, numerous studies have evidenced that GA producing microorganisms are categorized as plant growth-promoting rhizobacteria (Bottini et al., 2004).

Upon inoculation of plants with the biocontrol strain ST03, we observed a change in root architecture, mainly as an increase in lateral roots and root hairs and shortening of the root length (Figures 4A, 6A). Similar modes of action have been reported for plant growing rhizobacteria on roots (Dobbelaere et al., 1999). Apart from bacterial IAA biosynthesis, bacterial activation of, ACC (1-aminocyclopropane-1-carboxylate) deaminase is also responsible for plant growth promotion through reduction of ethylene (Spaepen and Vanderleyden, 2011).

Together with direct antagonism and the action on auxin and GA signaling, our findings indicate that the biocontrol strain ST03 slightly induces *PAL* (in SA pathway), *AOS*, and *LOX3* (in JA pathway) and PRs-related genes (*PR1*, *PR2*, *PR3*, and *PR10*) even in the absence of a pathogen. Prior studies have also shown that rhizobacteria can induce the archetypal defense pathways in plants related to SA and/or JA (Conn et al., 2008; Kurth et al., 2014; Tan et al., 2020; Vergnes et al., 2020). Notably, the accumulation of SA and JA correlate to the strong activation of the PRs-related genes *PR1*, *PR2*, *PR3*, and *PR10* using a tobacco-based model plant (Niki et al., 1998). *PR2* and *PR3* encoding glucanase and chitinase respectively, which are the two foremost antifungal components, play crucial roles in the plant defense system (Van Loon, 1997).

In addition, BXs and their derivatives such as 2,4–dihydroxy–1,4–benzoxazin–3–one (DIBOA), 2,4–Dihydroxy–7–methoxy–2H–1,4–benzoxazin–3(4H)–one (DIMBOA), and 6–methoxybenzoxazolin–3–one (MBOA) are the key defense secondary metabolites in maize against pathogens and insects (Niemeyer, 2009; Lanubile et al., 2017). Even though the biosynthesis pathways of such antimicrobial metabolites in maize have been well documented (Cotton et al., 2019), the study of the action of actinobacteria on the BXs biosynthesis pathways in maize remains poorly understood. In this current work, the *Streptomyces* strain ST03 did not induce the BXs biosynthesis-related genes, but when co-applied with *F. verticillioides*, a small amount of induction of *BX6*, *BX8*, and *BX9* was observed in the leaves. Ding et al. (2015) demonstrated that resistance of maize to *Bipolaris maydis*, a pathogen causing corn leaf blight, strongly correlates to up-regulation of BXs gene family (*BX1* and *BX8*). Also, BXs-dependent resistance of maize toward *Spodoptera littoralis*, a leaf feeder, has been reported (Maag et al., 2016). In agreement with Tran et al. (2021c), *F. verticillioides* induced the upregulation of the BXs genes in leaves of line *Bt/GT* NK7328. This finding might be helpful for one who wants to develop FER-resistant maize lines associated with these genes.

## Conclusion

Our data demonstrated the *Streptomyces* strain ST03 as a promising and effective biocontrol and plant growth-promoting candidate toward good management of *Fusarium* ear rot in maize. We uncovered two modes of action of strain ST03 against *F. verticillioides* comprising (1) direct antagonism and (2) modulating plant defense system via transient regulation of auxin signaling and archetypal defense pathways in plants. This strain could thus be used to develop a biofertilizer formulation toward sustainable agriculture.

## Data Availability Statement

The datasets presented in this study can be found in online repositories. The names of the repository/repositories and accession number(s) can be found below: https://www.ncbi.nlm.nih.gov/genbank/, MZ614615; https://www.ncbi.nlm.nih.gov/genbank/, MZ614616; https://www.ncbi.nlm.nih.gov/genbank/, MZ614619; and https://www.ncbi.nlm.nih.gov/genbank/, MZ614620.

## Author Contributions

TT: conceptualization, performing, formal analysis, writing, and original draft. MA: reviewing and editing. SD: supervision, reviewing, and editing. FD: supervision, reviewing, and editing. ME: supervision, reviewing, and editing. KA: supervision, conceptualization, reviewing, and editing. All authors contributed to the article and approved the submitted version.

## Conflict of Interest

The authors declare that the research was conducted in the absence of any commercial or financial relationships that could be construed as a potential conflict of interest.

## Publisher’s Note

All claims expressed in this article are solely those of the authors and do not necessarily represent those of their affiliated organizations, or those of the publisher, the editors and the reviewers. Any product that may be evaluated in this article, or claim that may be made by its manufacturer, is not guaranteed or endorsed by the publisher.

## Footnotes

1
https://cran.r-project.org/

## Supplementary Material

Supplementary Figure 1 **(A)** *In vitro* diffusion assay. **(B)** *In vitro* volatile assay using tryptic soy agar. **(C)** *In vitro* assay using tryptic soy broth. **(D)** *In vivo* assay on maize cobs. **(E)** Workflow diagram of *in planta* assay.

Supplementary Figure 2 *In vitro* inhibition efficacy (%) of four *Streptomyces* strains (ST02, ST03, ST07, and ST08) against six *Fusarium* species. The inhibition efficacy was calculated as a formula: (Rm – Rb)/Rm × 100 where Rm is a fungal radius of a mock control, and Rb is a fungal radius of a biocontrol treatment. Different letters by columns pinpoint a significant difference between treatments at each time point using an ANOVA test and a *post hoc* Turkey test at α = 0.05. Four cell suspensions of ST02, ST03, ST07, and ST08, respectively, and one 10-time-concentrated supernatant of the strain ST03 (Sup-ST03) were used. Water or tryptic soy broth were used as the mock controls. Four biological replicates were done per treatment. The experiment was repeated twice.

Supplementary Table 1 Primers of target genes and reference genes used in this study.
