# Supplementary figures and images for: Streptomyces Strains Promote Plant Growth and Induce Resistance Against Fusarium verticillioides via Transient Regulation of Auxin Signaling and Archetypal Defense Pathways in Maize Plants
Source: Front Plant Sci. 2021 Nov 25;12:755733. doi: 10.3389/fpls.2021.755733 (PMC8655691; doi:10.3389/fpls.2021.755733)

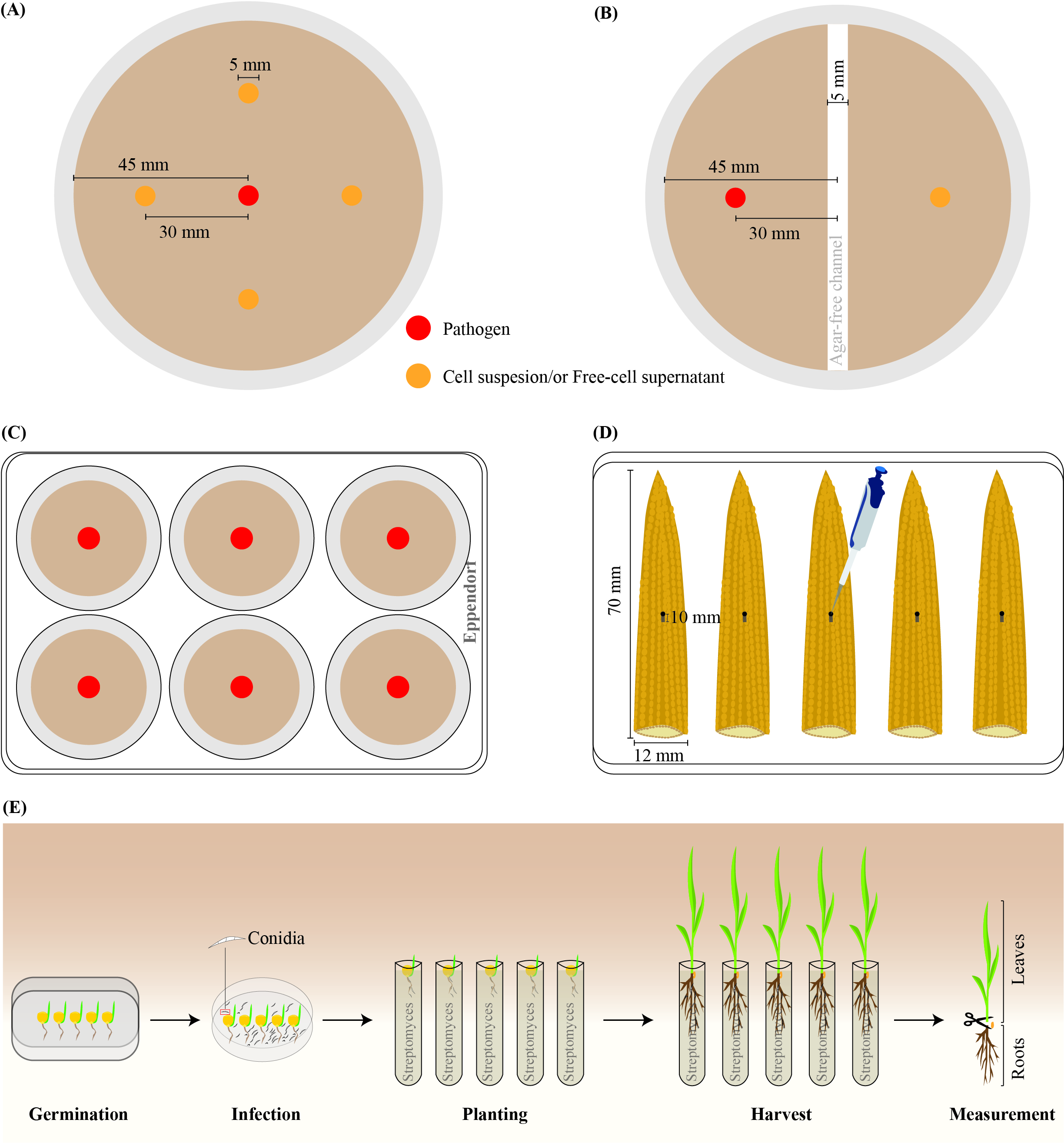

Supplement: Supplementary Figure 1 — (A) In vitro diffusion assay. (B) In vitro volatile assay using tryptic soy agar. (C) In vitro assay using tryptic soy broth. (D) In vivo assay on maize cobs. (E) Workflow diagram of in planta assay. [file Image_1.jpg]

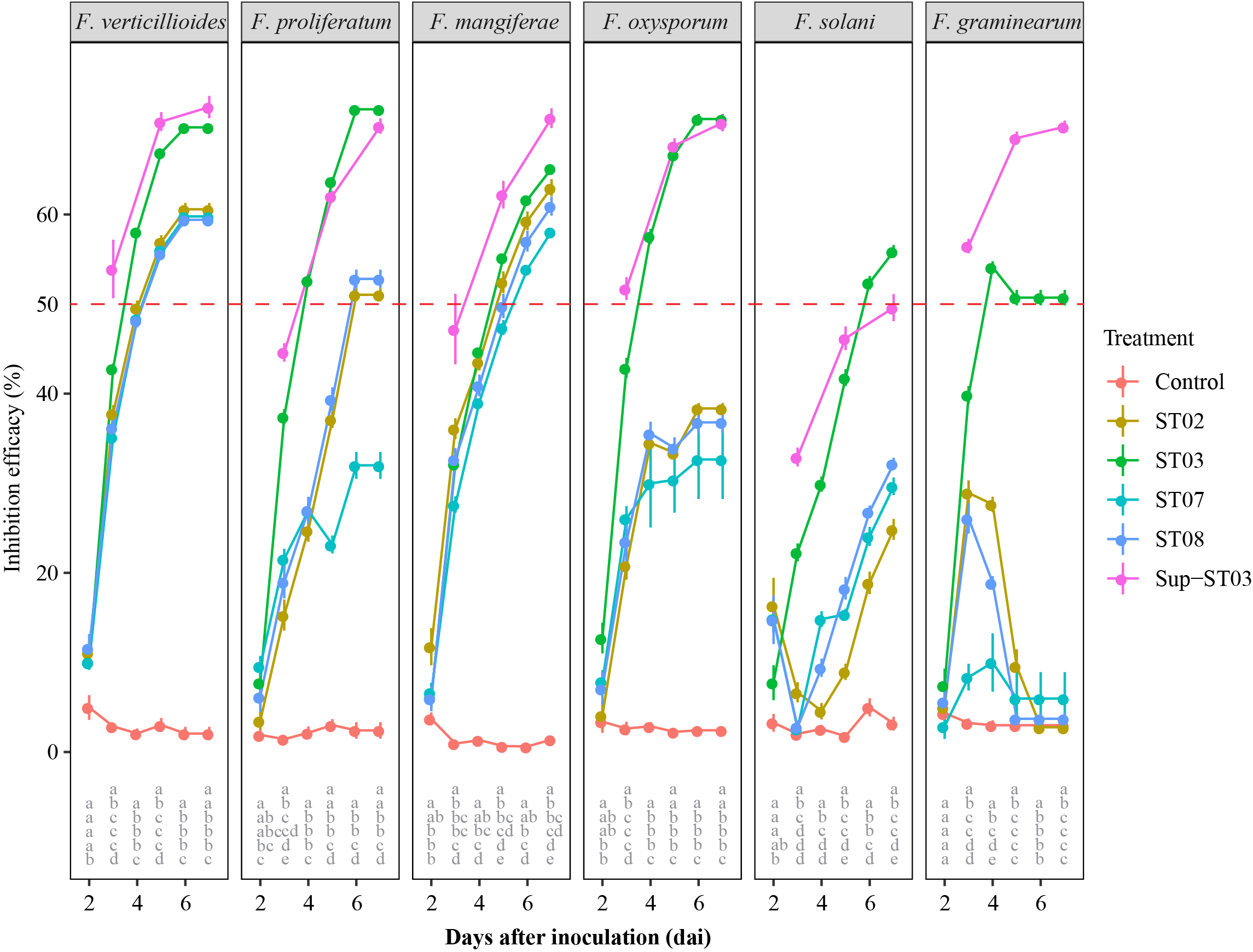

Supplement: Supplementary Figure 2 — In vitro inhibition efficacy (%) of four Streptomyces strains (ST02, ST03, ST07, and ST08) against six Fusarium species. The inhibition efficacy was calculated as a formula: (Rm – Rb)/Rm × 100 where Rm is a fungal radius of a mock control, and Rb is a fungal radius of a biocontrol treatment. Different letters by columns pinpoint a significant difference between treatments at each time point using an ANOVA test and a post hoc Turkey test at α = 0.05. Four cell suspensions of ST02, ST03, ST07, and ST08, respectively, and one 10-time-concentrated supernatant of the strain ST03 (Sup-ST03) were used. Water or tryptic soy broth were used as the mock controls. Four biological replicates were done per treatment. The experiment was repeated twice. [file Image_2.jpg]
