# Supplementary material for: Streptomyces Strains Promote Plant Growth and Induce Resistance Against Fusarium verticillioides via Transient Regulation of Auxin Signaling and Archetypal Defense Pathways in Maize Plants
Source: Front Plant Sci. 2021 Nov 25;12:755733. doi: 10.3389/fpls.2021.755733 (PMC8655691; doi:10.3389/fpls.2021.755733)
Supplement: Supplementary Table 1 — Primers of target genes and reference genes used in this study. [file Table_1.docx]

**Table S1.** Primers of target genes and reference genes used in this study.

| **Gene** | **Gene annotation** | **Forward primer (5’ – 3’)** | **Reverse primer (5’ – 3’)** | **Reference** |
| --- | --- | --- | --- | --- |
| *LOX3* | Lipoxygenase 3 | CGTGTAAACGGGAAGAGAGC | CCAATGATTGCAACAAGCAC | Tzin *et al.*, 2017 |
| *LOX10* | Lipoxygenase 10 | GACATCCTCTCGTCGCACTC | ATGAACCCCTCGATCTCCTT |  |
| *BX6* | 2-oxoglutarate-dependent oxygenase 6 | GAGTGCATGCAGAACCTGAA | GCAGGAGGATGGTGAAGAAG |  |
| *PAL* | Phenylalamine ammonia-lyase | aagaaggtgaacgagctgga | gttgtcgttcacggagttga | Ding *et al.*, 2015 |
| *PR10* | Pathogenesis-related protein 10 | gtcatgccgttcagcttcat | tgttcttgcactcgacttg |  |
| *AOS* | Allene oxide synthase | acctgttcacgggcacctac | cgaggagcgaggagaagttg |  |
| *BX8* | 2-oxoglutarate-dependent oxygenase 8 | gatacctgccggtgagagag | gggaacgtgtggaagatgag |  |
| *BX9* | 2-oxoglutarate-dependent oxygenase 9 | gcaacatgaggtacgtgtgc | gcagcgatcttgaattcctt |  |
| *ABI* | Homology to glycin-rich protein | gcgagatcctcgactccaag | gggcttggttaacggtgatg |  |
| *PR1* | Pathogenesis related protein 1 | GAACTCGCCGCAGGACTAC | GAGCCCCAGAAGAGGTTCTC | Lanubile *et al.*, 2010 |
| *PR2* | 1,3-β-glucanse | GCGCAGACCTACAACCAGA | GGAGAAATTGATGGGGTACG |  |
| *PR3* | Chitinase | GGCTCTACGCCTACGTCAAC | GATGGAGAGGAGCACCTTGA |  |
| *AUX1* | Auxin transporter-like protein 1 | CATCTGGTTCCTCGCCATCATCTTC | TGACGTAAGCGCCTGTCCACCCT | Zhang *et al.*, 2019 |
| *ARF1* | Auxin response factor 1 | CTTGTGCTGGGCCGTTGGTC | GGCTGGTGGGCTCACTTTGC |  |
| *ARF2* | Auxin response factor 2 | CACCGACTACGGCGAACTCC | GCCACCAAGAGCAAACCACC |  |
| *AN1* | CPP synthase, anther ear 1 | GTGACATTTCGGAAGACAAGA | CGTCCAGCACAAATCTCAATA |  |
| *EF-1α* | Elongation factor 1 alpha | TGGGCCTACTGGTCTTACTACTGA | ACATACCCACGCTTCAGATCCT | Lin *et al.*, 2014 |
| *β-TUB* | Beta tubulin | CTACCTCACGGCATCTGCTATGT | GTCACACACACTCGACTTCACG |  |
| *FVer* | Marker gene for detection of *Fusarium verticillioides* | CGTTTCTGCCCTCTCCCA | TGCTTGACACGTGACGATGA | Nicolaisen *et al.*, 2009 |
| *recA* | Recombinase A | ACAGATTGAACGGCAATTCG | ACCTTGTTCTTGACCACCTT | Guo *et al.*, 2008 |
